# Generalizability of GWA-Identified Genetic Risk Variants for Metabolic Traits to Populations from the Arabian Peninsula

**DOI:** 10.3390/genes12101637

**Published:** 2021-10-18

**Authors:** Prashantha Hebbar, Mohamed Abu-Farha, Jehad Abubaker, Arshad Mohamed Channanath, Fahd Al-Mulla, Thangavel Alphonse Thanaraj

**Affiliations:** 1 Department of Genetics and Bioinformatics, Dasman Diabetes Institute, P.O. Box 1180, Dasman 15462, Kuwait; prashantha.hebbar@dasmaninstitute.org (P.H.); arshad.channanath@dasmaninstitute.org (A.M.C.); 2 Department of Biochemistry and Molecular Biology, Dasman Diabetes Institute, P.O. Box 1180, Dasman 15462, Kuwait; mohamed.abufarha@dasmaninstitute.org (M.A.-F.); jehad.abubaker@dasmaninstitute.org (J.A.); 3 Research Division, Dasman Diabetes Institute, Dasman 15462, Kuwait

**Keywords:** risk loci, metabolic traits, GWAS, transferability of risk loci, population diversity, Arab ancestry

## Abstract

The Arabian Peninsula, located at the nexus of Africa, Europe, and Asia, was implicated in early human migration. The Arab population is characterized by consanguinity and endogamy leading to inbreeding. Global genome-wide association (GWA) studies on metabolic traits under-represent the Arab population. Replicability of GWA-identified association signals in the Arab population has not been satisfactorily explored. It is important to assess how well GWA-identified findings generalize if their clinical interpretations are to benefit the target population. Our recent study from Kuwait, which performed genome-wide imputation and meta-analysis, observed 304 (from 151 genes) of the 4746 GWA-identified metabolic risk variants replicable in the Arab population. A recent large GWA study from Qatar found replication of 30 GWA-identified lipid risk variants. These complementing studies from the Peninsula increase the confidence in generalizing metabolic risk loci to the Arab population. However, both the studies reported a low extent of transferability. In this review, we examine the observed low transferability in the context of differences in environment, genetic correlations (allele frequencies, linkage disequilibrium, effect sizes, and heritability), and phenotype variance. We emphasize the need for large-scale GWA studies on deeply phenotyped cohorts of at least 20,000 Arab individuals. The review further presents GWA-identified metabolic risk variants generalizable to the Arab population.

## 1. Introduction

Over the past few years, a multitude of global genome-wide association (GWA) studies have identified genetic risk variants associated with metabolic traits and related disorders. Efforts to translate GWAS findings into polygenic risk scores (PRS) across populations to decipher their clinical interpretation are gaining momentum [1,2]. Our recent examination of GWAS Catalog [3] against 313 search terms relating to four classes of metabolic traits (namely anthropometry, glycemia, lipid, and blood pressure) found 7668 genetic variants from ~4000 genes associated with metabolic traits [4]; association signals involving 4746 (i.e., 62%) of the 7668 variants were at genome-wide significance (*p*-values of ≤5.0 × 10^−8^). A majority of these studies were performed on populations of European, East Asian, and African ancestries. Arab populations from the Middle East are among the most underrepresented in genetic studies [5,6,7,8]. Factually, 88.65% of GWA studies, summarized in the GWAS Catalog, were Europeans, while only 7.02% were Asians and 4.33% Africans, Latin, and other populations [9] (www.gwasdiversitymonitor.com, accessed on 21 July 2021). Applicability of clinical translation of such GWA-identified risk loci and PRS to ethnic populations, under-represented in global studies, depends on the generalizability of the underlying association signals to the target populations.

By virtue of being situated between Africa, Europe, and South Asia, the Arabian Peninsula forms an important region in the history of early human migrations and admixtures [5,6,8]. We and other researchers have illustrated that major sources of ancestry forming the modern Arab population are from sub-Saharan/Western Africa and from West Eurasia [8,10,11,12]. The region had several humid periods resulting in a “green Arabia”, which facilitated human dispersals and migrations [8]. The onset of the current desert climate is thought to have started around six thousand years ago [13]. Eventually, the inhabitants of the Peninsula region adapted to the hot and dry environment. The adaptation and natural selection shaped the extant human populations of the Arabian Peninsula region [8,14]; for example, we demonstrated that a haplotype overlapping *TNKS* showed strong signals of positive selection in the Arab cohort and proposed that this haplotype under selection potentially conferred a fitness advantage to the Kuwaiti ancestors for surviving in the harsh environment while posing a major health risk to present-day Kuwaitis [14].

The Arab population is characterized by unique features such as large families, consanguinity, endogamy, and first-cousin marriages, which have resulted in creation of inbreeding communities. Such inbreeding communities are expected to have increased homozygosity at-risk variants for both monogenic and polygenic diseases as well as an accumulation of deleterious recessive alleles in the gene pool; our previous GWA study under a genetic model based on the recessive mode of inheritance pinpointed 16 novel risk variants associated with plasma TG levels in Arab individuals from Kuwait [15,16]. Familial aggregation of hypercholesterolemia [17], type 2 diabetes [18,19,20,21], and type 1 diabetes [22] is prominent among Arabs. Further, the exceptional growth in prosperity in the Arabian Peninsula during the rich post-oil era brought rapid changes in lifestyles (such as urbanization, dietary changes, low levels of physical activity, and high levels of sedentary behavior) leading to chronic metabolic disorders [23]. These rapid lifestyle changes are expected to have an impact on gene–environment interactions; several diet–genetics–disease relationships in the region have been discussed as contributing to the increased prevalence of metabolism disorders and micronutrient deficiencies [24]. Furthermore, the Arab populations appear to have a higher genetic risk for metabolic disorders such as diabetes—for example, a study on Arab immigrants in the USA found that they had a higher risk of type 2 diabetes than native inhabitants [25]. Another study of Middle Eastern immigrants in Sweden found that the immigrants had a two- to threefold higher risk of type 2 diabetes than native Swedes [26]. We have discussed, in our earlier publication [27], that combinations of such lifestyle changes, gene–environment interactions, and genetic predispositions have probably led to the dramatic increase in the prevalence of obesity, diabetes, and dyslipidemia in Arabs.

Replicability of GWA-identified association signals for metabolic traits by global studies to Arab population has not been explored to satisfaction. It is important to assess how well the GWA-identified risk loci generalizes, if a target population is to benefit from clinical interpretation of global GWA findings.

## 2. GWA Studies for Metabolic Traits on Arab Populations

A literature review by us in 2019 [27] reported that only 25 GWA-identified risk loci for metabolic traits have been replicated, largely by targeted genotyping studies, in Arab populations. Our recent genome-wide imputation and meta-analysis study from Kuwait [4] used a cohort of 2732 Arab individuals and observed that association signals involving only 304 (6.4%) of the 4746 metabolic risk variants identified at genome-wide significance in global GWA studies were replicable in the Kuwaiti cohort. These 304 variants are from 151 distinct genes (Supplementary Table S1). The GWA studies observed 178 of these 304 GWA-identified risk variants in more than one population. The GWA study cohorts for these 304 variants were largely of European ancestry (i.e., in 260 of the 304 variants). Many of these transferable GWA-identified signals were observed in the Kuwaiti cohort at borderline significance, suggestive of association. In the same study [4], we further performed power calculations that considered effect sizes of GWA-identified risk variants and their allele frequencies in the Kuwaiti cohorts and projected a sample size of at least 10,000 to observe these 304 GWA-identified association signals for metabolic traits at genome-wide significance in the Arab population. The study further projected a necessary sample size of 20,000 in order to observe the other GWA-identified association signals in the Arab population.

In a recent study, Thareja et al. [28] performed genome-wide association tests to delineate risk variants for 45 clinically relevant traits using a discovery set of whole-genome sequences of 6218 Qatari individuals. The examined traits included two (namely anthropometry and lipid) of the four classes of metabolic traits examined in our study [4]. Though Thareja et al. used a large sample size of 6218, nearing two-thirds of the 10,000 projected by our study, they observed only four GWA-identified association signals relating to anthropometric traits and 26 GWA-identified association signals relating to lipid traits at genome-wide significance. One of these four anthropometric trait association signals and 22 of the 26 lipid trait association signals were observed in our study [4] (Table 1). These 23 association signals for lipid traits comprised 18 distinct variants from 15 genes (Table 2); 10 of these 18 distinct variants are “low-frequency” (MAF < 5%) variants in one of the examined populations while “common” (MAF > 5%) in other populations.

## 3. Generalizability of GWA-Identified Association Signals in Arab Populations

The results presented by the above-mentioned two studies [4,28] from Kuwait and Qatar indicate that the assessment of generalizability of GWA-identified association signals in the Arab population is still an “open” question. Though it is possible that the limited sample sizes and differences in study designs may contribute to the observed low extent of transferability, the role of differences in factors such as phenotypic variance due to unique environmental conditions, allele frequencies, and linkage disequilibrium profiles cannot be ruled out. Thareja et al. [28], by way of using variants with minor allele frequency (MAF) >1%, derived reasonable heritability (h^2^) values for obesity traits (height = 0.59; BMI = 0.31) and lipid traits (TC = 0.22; HDL-C = 0.41; LDL-C = 0.21; TG = 0.31) in the Qatari cohort. Further, they demonstrated a high overall correlation in heritability with European (r^2^ = 0.81) populations compared to a low, yet reasonable, correlation with African (r^2^ = 0.44) populations, suggesting that much of the association signals seen in Europeans are transferable to Arabs. However, the heritability values for obesity and lipid traits, when individually examined, were significantly lower in the Qatari cohort compared to Europeans, suggesting that much of the heritability of obesity and lipid traits is still not explained by the study. Since a great proportion of phenotypic variance for complex traits is contributed by rare variants (MAF < 1%) [29], an effective study of heritability requires a further large cohort. These variations in heritability also warrant the need for more Mendelian Randomization studies to pinpoint the environmental factors causally linked to trait associations.

In our study from Kuwait [4], we observed that only those GWA-identified variants with larger effect sizes replicate well in the Arab population; failure to replicate the variants with small effect sizes could be due to the modest sizes of our study cohorts. Thareja et al. [28] found significant differences in both effect size and allele frequency of variants associated with replicated risk loci and emphasized the need for further large GWAS to determine accurate PRS in the Arab population. Complex metabolic disorders are influenced by multiple common genetic variants with small effect size; hence, meaningful polygenic risk scores (PRS) are derived by inspecting the cumulative effect of multiple variants. Such multiple genetic variants used to build PRS can differ in allele frequencies across populations due to reasons such as natural selection and population expansion leading to adaptation to local environmental factors. A recent study from Iran [30] found multiple T2D-risk SNPs that were significantly depleted or enriched in at least one of the five populations of the 1000 Genome Project (African, American, East Asian, European, and South Asian) as well as the Iranian population. They further found that a PRS built using the enriched risk alleles in Iran was significantly associated with type 2 diabetes incidence in their longitudinal cohort study. The global GWA studies are highly Eurocentric. As a result, PRS developed using risk variants identified through such global studies do not predict individual risk accurately in non-Europeans [31]. To realize the full and equitable potential of PRS in ethnic populations such as Arabs, there is a need to prioritize greater diversity in global genetic studies.

Differences in linkage disequilibrium (LD) patterns among populations appear to play a role in predictive differences [32,33]. A GWA variant strongly associated with a trait in one population may not have a detectable association in another, as the LD with the (unknown) causal variant may be much weaker [34]. LD for each Middle East population decayed faster than European and East Asian populations but slower than African populations [35]. The study by Thareja et al. [28] showed marked differences in linkage disequilibrium and allele frequencies among the European, East Asian, and Qatari populations. We found in our earlier study that, though the LD decay patterns seem to exhibit similar rates across the populations, the conservation values are different at any given distance—the population subgroups from Kuwait showed lower conservation values than the European French population [12].

Recently, an interesting framework of the omnigenic model has been proposed [36,37] to explain the observed low transferability of polygenic scores and the variations in effect sizes across populations. The model explains how the interaction network comprising ‘core’ genes of GWAS findings and ‘peripheral’ (to the core) genes (participating in the pathway) ultimately leads to causality of phenotype through gene × environment interactions. The Arab population went through ‘rapid’ lifestyle changes in the post-oil era. Further, the two populations differ considerably in climate conditions. Even with consistency in effect sizes between European and Arab populations, the effect of ‘core’ genes on phenotype via the ‘peripheral’ gene network can differ because of differences in gene × environment interactions; thus, the predictive power of polygenic scores can differ substantially across these two population groups. On the other hand, heterogeneity in effect size (or even direction) at transferable GWA loci to the Arab population could be due to differences in LD structure and allele frequency. Often, the direct estimates of genetic correlations of cross-populations are less than one. Although the difference in the contribution of ‘core’ genes to the loss of variance at PRS level is small, much of the variance loss is likely due to differences in LD, allele frequency, and causal effect by gene × environment of ‘peripheral’ genes [36]. Hence, the predictive power of polygenic risk scores decreases more severely than what would be expected for given differences in allele frequency and LD structure at ‘core’ genes alone.

## 4. ‘Novel’ Risk Variants for Lipid Traits in Arab Populations

Thareja et al. [28] identified a novel variant (rs376997679, located downstream of *CADM1*) associated with TG. Though it is a common variant (MAF = 5.92%) in the Qatari cohort, it is rare in continental populations. Rare variants from *CADM1* have been recently implicated in anorexia nervosa, a subtype of eating disorder [38]. Similarly, in our study from Kuwait [4], we found a novel locus rs76018028 from [LOC105377613-LOC105377614], along with 25 accompanying LD variants, associated with low HDL levels. Further, in an earlier work [16], we identified a few more novel variants (from genes such as *CDK12-NEUROD2*, *RPS6KA1*, *LAD1*, *PGAP3*, and *CERK*) for lipid traits in the Arab population. Identification of novel risk loci for lipid traits in Arabs is important given the unique phenotypes: (i) the high prevalence of metabolic syndrome, diabetes, familial hypercholesterolemia, and consanguineous marriages in the Middle East region resulted in a pattern of dyslipidemia that is different from elsewhere in the world [39]; (ii) the Dyslipidemia International Study (DYSIS) indicated that while 61.8% of statin-treated participants from the Middle East cohort missed their therapeutic LDL-cholesterol goal [40], only up to 48.2% of statin-treated participants from the European and Canadian cohorts missed the goal [41].

## 5. Future Directions

By way of discussing two recent GWA studies of sample sizes of 6218 and 2732 individuals from Qatar and Kuwait, respectively, we reviewed the transferability of GWA genetic findings to the Arab populations in the Middle East. We propose a sample size of at least 20,000 Arab individuals to enable a more comprehensive assessment of the translational value of genetic and genomic findings from global GWA studies on populations, typically dominated by European ancestry, to the Arab population. The genetics of the Arab population in the Peninsula has been largely driven by consanguinity and inbreeding. A combination of rapid lifestyle changes in the rich post-oil era, gene–environment-disease interactions, and the inherent genetic predispositions has shaped the observed high prevalence of metabolic disorders in the region. The assessment and delineation of the exact translatable genetic findings represent a foundation for transferring the implementation of precision medicine to the Middle East.

Aside from the need for a larger sample size, it is becoming increasingly evident that deep phenotyping through the utilization of gold standard, scalable metabolic techniques, such as oral glucose tolerance test, liver imaging, and bioelectrical impedance (that measures body fat composition distributions), is essential. For example, using a euglycemic insulin clamp technique, Hassoun et al. [42] demonstrated that a small increase of 1.2 units from normal BMI in Arabs as compared to Mexican Americans could be associated with high insulin resistance. Additionally, lean Arab participants compared to Mexican Americans had a more severe level of insulin resistance where Arabs had a 28% less total body glucose disposal compared to lean Mexican Americans. Additionally, Arabs had a much lower total body glucose disposal rate across all BMI levels compared to Mexican Americans. The presence of such a severe insulin resistance in lean Arab individuals with impaired glucose tolerance suggests that it is caused by a combination of genetic and environmental factors. Such ethnic differences can only be identified through utilizing sensitive techniques that allow us to pinpoint metabolic abnormalities. Additionally, increased utilization of cutting-edge imaging techniques will also allow us to better understand the fat tissue distribution and establish its association with genetic variants within various ethnic groups. In another example, Ji et al. [43], by way of using MRI data quantifying body fat distribution in combination with GWAS data, showed that while 14 alleles were associated with higher BMI and higher body fat percentage, they were also associated with lower risk for type 2 diabetes, heart disease, and hypertension. They concluded that carriers of these alleles, particularly in *PPARG*, *GRB14*, and *IRS1* genes, had higher subcutaneous fat and lower ectopic fat accumulation. Both these two examples demonstrate the need for deep phenotyping to properly establish the genetic associations with metabolic factors beyond the basic clinical traits. One of our earlier works on the obesity gene of *FTO* [44], by way of demonstrating that an *FTO* variant rs1421085 associates with total body water and soft lean mass through interaction with ghrelin and apolipoproteins in the Arab population, illustrates the insight that deep phenotyping can bring to transferability of GWA-identified association signals to the ethnic population.

In summary, transferability of GWAS findings and their clinical interpretation to diverse population groups is an important issue in the community. The Arabian Peninsula forms an important region in the history of early human migrations and admixtures. The Arab population is characterized by large families, consanguinity, endogamy, inbreeding, and familial aggregation of metabolic disorders. Notable GWA studies, including ours, of modest cohort sizes from the Arab region demonstrated the transferability at genome-wide significance of a minor proportion of the GWA-identified metabolic risk variants. These studies further identified few novel risk variants at genome-wide significance. A sample size of 20,000 has been estimated as a good size to detect a large proportion of established GWA variants in the Arab population. The review highlights the linkages between these studies from the region and potential for transferability:

(a) Strength of LD between a GWA variant and the causal variant can vary across populations; hence, the GWA variant may not be equally detectable across population groups. Marked differences in LD have been observed among the European, East Asian, and Arab populations; hence, population-specific differences in LD need to be considered while assessing transferability.
(b) A proper assessment of transferability requires considering gene–environment interacting network models that include gene × environment interactions (involving not only the core genes of GWAS findings but also their peripheral genes) in determining the causality of phenotype. In contrast to Europeans, the Arab population went through recent ‘rapid’ lifestyle changes due to wealth from the post-oil era.
(c) PRS for metabolic disorders is derived by the cumulative effect of multiple genetic variants; differences in allele frequencies at such variants across populations—due to reasons such as natural selection, population expansion, and adaptation to local environmental factors—make the PRS not readily transferable.
(d) Finally, the review emphasizes the need for deeply phenotyped cohorts to properly assess the transferability and to get new insights into established association signals

## 6. Conclusions

In conclusion, the risk factors unique to Arabs, offer a unique challenge compared to other counterpart populations, in terms of choice of genetic models and phenotypes, analysis designs and strategies. These two recent GWA studies have enlightened a need for large size cohorts and holistic data analysis approaches to reveal yet unidentified genetic signatures for metabolic disease traits in Arab population. This review attempts to summarize the findings in terms of novelty and transferability of genetic associations, the grasp of the situation and plausible challenges to be addressed by the metabolic disease research community.

## Figures and Tables

**Table 1:** GWA-identified association signals for lipid traits occurring in both the Kuwaiti and Qatari Cohorts.

Qatar							Kuwait				
Trait	SNP	Gene	A1	A2	β	*p*-Value	A1	A2	Zscore	*p*-Value	Trait
BMI	rs17817449	*FTO*	T	G	0.095	2.52 × 10^8^	T	G	2.852	0.0043	WT
									2.842	0.0044	DBP
									−3.83	0.0001	HDL
									3.466	0.0005	TG
									3.249	0.0011	*BMI*
									2.865	0.0041	WC
									2.276	0.0228	SBP
									2.14	0.0323	NON_HDL
HDL-C	rs74869266	*LPL*	A	C	0.236	2.65 × 10^10^	A	C	2.002	0.0452	LDL
HDL-C	rs1077834	*LIPC*	T	C	0.109	3.51 × 10^8^	T	C	2.807	0.005	logFPG
HDL-C	rs708272	*CETP*	G	A	0.192	1.04 × 10^28^	A	G	−6.285	3.28 × 10^10^	***HDL***
									2.074	0.0381	TG
									2.06	0.0393	NON_HDL
HDL-C	rs7499892	*CETP*	C	T	−0.198	9.88 × 10^19^	T	C	6.424	1.33 × 10^10^	***HDL***
									−2.389	0.0168	HT
LDL-C	rs12740374	*CELSR2*	G	T	−0.192	3.83 × 10^15^	T	G	4.412	1.02 × 10^5^	TC
									4.939	7.86 × 10^7^	NON_HDL
									4.39	1.13 × 10^5^	***LDL***
LDL-C	rs1800481	*APOB*	G	A	−0.175	1.16 × 10^12^	A	G	4.191	2.78 × 10^5^	***LDL***
									4.068	4.74 × 10^5^	NON_HDL
									3.672	0.0002	TC
LDL-C	rs111989435	*SMARCA4*	A	G	−0.192	2.42 × 10^15^	A	G	−4.937	7.93 × 10^7^	***LDL***
									−4.13	3.63 × 10^5^	NON_HDL
									−4.08	4.51 × 10^5^	TC
									2.061	0.0393	DBP
LDL-C	rs111234557	*MAU2*	C	G	−0.207	8.60 × 10^9^	C	G	2.144	0.0319	HDL
									−5.499	3.83 × 10^8^	NON_HDL
									−4.901	9.52 × 10^7^	TG
									−4.787	1.70 × 10^6^	TC
									−3.492	0.0004	***LDL***
									2.857	0.0042	logFPG
									2.308	0.021	logHbA1C
LDL-C	rs429358	*APOE*	T	C	0.23	4.22 × 10^12^	T	C	−2.226	0.026	logFPG
									2.122	0.0338	TG
									−2.849	0.0043	HDL
LDL-C	rs7412	*APOE*	C	T	−0.566	6.27 × 10^29^	T	C	2.314	0.0206	SBP
									4.233	2.31 × 10^5^	***LDL***
									3.547	0.0003	NON_HDL
									3.077	0.002	TC
TG	rs1260326	*GCKR*	C	T	0.122	4.11 × 10^12^	T	C	3.292	0.0009	LDL
									−4.833	1.35 × 10^6^	***TG***
									2.545	0.0109	HT
TG	rs33951980	*MLXIPL*	C	T	−0.112	2.09 × 10^8^	T	C	3.595	0.0003	***TG***
TG	rs10892002	*AP000770.1*	G	C	0.098	2.25 × 10^8^	C	G	2.5	0.0124	TC
									2.126	0.0335	LDL
									−2.114	0.0345	WC
TG	rs6589570	*APOA5*	T	A	0.195	4.19 × 10^16^	A	T	2.16	0.0307	LDL
									−4.766	1.88 × 10^6^	***TG***
TG	rs5095	*APOA4*	A	G	−0.133	5.18 × 10^12^	A	G	−2.162	0.0306	***TG***
TG	rs111234557	*MAU2*	C	G	−0.184	4.72 × 10^8^	C	G	−5.499	3.83 × 10^8^	NON_HDL
									−4.901	9.52 × 10^7^	***TG***
									−4.787	1.70 × 10^6^	TC
									−3.492	0.0004	LDL
									2.857	0.0042	logFPG
									2.308	0.021	logHbA1C
									2.144	0.0319	HDL
TCH	rs7528419	*CELSR2*	A	G	−0.175	6.51 × 10^13^	A	G	−5.046	4.51 × 10^7^	NON_HDL
									−4.547	5.45 × 10^6^	***TC***
									−4.546	5.47 × 10^6^	LDL
TCH	rs1800481	*APOB*	G	A	−0.162	4.75 × 10^11^	A	G	4.191	2.78 × 10^5^	LDL
									4.068	4.74 × 10^5^	NON_HDL
									3.672	0.0002	***TC***
TCH	rs8106503	*LDLR*	T	C	−0.168	1.22 × 10^12^	T	C	−5.114	3.15 × 10^7^	LDL
									−4.204	2.62 × 10^5^	***TC***
									−4.196	2.71 × 10^5^	NON_HDL
									2.232	0.0256	DBP
									1.996	0.0459	SBP
TCH	rs111234557	*MAU2*	C	G	−0.238	3.54 × 10^11^	C	G	−5.499	3.83 × 10^8^	NON_HDL
									−4.901	9.52 × 10^7^	TG
									−4.787	1.70 × 10^6^	***TC***
									−3.492	0.0004	LDL
									2.857	0.0042	logFPG
									2.308	0.021	logHbA1C
									2.144	0.0319	HDL
TCH	rs429358	*APOE*	T	C	0.203	7.69 × 10^11^	T	C	−2.849	0.0043	HDL
									−2.226	0.026	logFPG
									2.122	0.0338	TG
TCH	rs7412	*APOE*	C	T	−0.422	6.52 × 10^17^	T	C	2.314	0.0206	SBP
									4.233	2.31 × 10^5^	LDL
									3.547	0.0003	NON_HDL
									3.077	0.002	***TC***

The same trait—variant associations among Qatar and Kuwait is highlighted with bold and italics in list of traits. Thareja et al. [28] observed four GWA-identified association signals in the Qatari cohort relating to anthropometric traits at genome-wide significance, which he Qatari cohort relating to lipid traits at genome-wide significance, which included 22 identified in the Kuwaiti meta-analysis cohort [4]; 17 of these 22 associations were with the same exact lipid trait in the Kuwaiti cohort; of the remaining five, four were observed with related lipid traits; and one with a related metabolic trait (FPG).

**Table 2:** List of SNPs forming GWA-identified association signals, for lipid traits, generalizable to both the cohorts of Kuwaiti and Qatari individuals.

SNP	Consequence	Gene	MAF						
			Qatar	Kuwait	Continents				
					AFR	AMR	EAS	EUR	SAS
**rs1077834**	**downstream**	***LIPC***	**0.23**	0.22	0.57	0.43	0.42	0.21	0.31
rs10892002	upstream	*AP000770.1*	0.45	0.43	0.22	0.33	0.15	0.43	0.43
rs111234557 ^@^	intron	*MAU2*	0.07	0.08	***0.02***	0.11	0.18	0.11	0.16
rs111989435 ^@^	intergenic	*SMARCA4*	0.17	0.17	0.19	0.10	***0.02***	0.12	0.07
rs1260326	Missense (L446P)	*GCKR*	0.42	0.47	0.09	0.36	0.48	0.41	0.20
rs12740374 ^@^	downstream	*CELSR2*	0.17	0.16	0.25	0.20	***0.04***	0.21	0.26
rs7528419 ^@^	downstream		0.17	0.16	0.27	0.20	***0.04***	0.21	0.26
rs17817449	intron	*FTO*	0.47	0.44	0.38	0.25	0.17	0.41	0.29
rs1800481 ^@^	upstream	*APOB*	0.16	0.18	0.29	0.13	***0.00***	0.19	0.11
rs33951980 ^@^	intron	*MLXIPL*	0.24	0.15	0.06	***0.04***	0.12	0.12	0.08
rs429358	downstream	*APOE*	0.08	0.09	0.27	0.10	0.09	0.16	0.09
rs7412 ^@^	Missenseandrei (R176C)	*APOE*	***0.03***	0.06	0.10	0.05	0.10	0.06	***0.04***
rs5095 ^@^	intron	*APOA4*	0.28	0.23	0.09	0.11	***0.00***	0.18	0.12
rs6589570	intergenic	*APOA5*	0.15	0.17	0.11	0.23	0.25	0.18	0.36
rs708272	intron	*CETP*	0.39	0.40	0.25	0.46	0.38	0.43	0.45
rs7499892	intron	*CETP*	0.17	0.19	0.41	0.22	0.16	0.21	0.22
rs74869266 ^@^	intergenic	*LPL*	0.06	0.06	***0.01***	0.05	0.03	0.11	0.06
rs8106503 ^@^	downstream	*LDLR*	0.18	0.18	0.30	0.17	***0.04***	0.11	0.14

**^@^**, These 10 risk variants are low-frequency variants (MAF < 5%) (indicated by bold and italics font) in one of the examined populations, while they are “common” variants in other populations.

## Data Availability

There are no new data presented in this paper. Please see Hebbar et al. [4] and Thareja et al. [28] for original data. The list of 304 metabolic risk variants from GWAS transferable to the Arab population is given as Supplementary Table S1.

## Supplementary Materials

The following are available online at https://www.mdpi.com/article/10.3390/genes12101637/s1, Supplementary Table S1. List of the 304 GWA-identified metabolic risk variants transferable to Arab population from Kuwait.
