# Supplementary material for: Generalizability of GWA-Identified Genetic Risk Variants for Metabolic Traits to Populations from the Arabian Peninsula
Source: Genes (Basel). 2021 Oct 18;12(10):1637. doi: 10.3390/genes12101637 (PMC8535608; doi:10.3390/genes12101637)
Supplement: Supplementary file 1 [file genes-12-01637-s001.zip › genes-1370066-supplementary/genes-1370066-suppl.pdf]

Supplementary Table S1. List of the 304 GWA-identified metabolic risk variants transferable to Arab population from Kuwait.

Variant (hg19)	Populations in which the association of the variant was identified	Replication Count	RSID	Gene
1:177889480	European,Indian,Korean,AfricanAmerican,Jamaican,Filipino,African American/Afro-Caribbean,Hispanic/Latino,Nigerian,Sub-SaharanAfrican,Hispanic,Seychelles,SouthAsian,Japanese,African,Seychellois,EastAsian,AfricanBritish	18	rs543874	LOC101928778 - SEC16B
11:116648917	European,Indian,AfricanAmerican,Hispanic,Mexican,SaudiArabian,LatinAmerican/Hispanic,Mylopotamos,Pomak,EastAsian,Chinese,Yoruban,Filipino	13	rs964184	ZNF259
1:96924097	European,AfricanAmerican/Afro-Caribbean,Indian,Filipino,Hispanic/Latino,AfricanAmerican,Hispanic,Seychelles,SouthAsian,Japanese,EastAsian,AfricanBritish	12	rs11165643	EEF1A1P11 - LOC105378866
2:632348	European,AfricanAmerican/Afro-Caribbean,Indian,Filipino,Hispanic/Latino,AfricanAmerican,Hispanic,Seychelles,SouthAsian,Japanese,EastAsian,AfricanBritish	12	rs13021737	LOC105373352 - TMEM18
16:56993324	European,Indian,NorthernFinnish,AfricanAmerican,AfricanAmerican/Afro-Caribbean,Japanese,HanChinese,Korean,EastAsian,British,Chinese	11	rs3764261	CETP
16:81534790	European,AfricanAmerican,Filipino,EastAsian,AfricanAmerican/Afro-Caribbean,SouthAsian,Seychellois,Indian,Hispanic/Latino,Chinese,Japanese	11	rs2925979	CMIP
6:163033350	AfricanAmerican,European,Filipino,Indian,AfricanAmerican/Afro-Caribbean,Hispanic/Latino,Hispanic,Seychelles,SouthAsian,EastAsian,AfricanBritish	11	rs13191362	PARK2
2:27730940	European,Indian,Orcadian,Hispanic,Afro-Caribbean,Thai,FrenchCanadian,Japanese,Mexican,EastAsian,Chinese	11	rs1260326	GCKR

2:56096892	European,Japanese,EastAsian,AfricanAmerican,Filipino,Indian,AfricanAmerican/Afro-Caribbean,Hispanic/Latino,SouthAsian,Seychellois	10	rs3791679	EFEMP1
13:79580919	European,AfricanAmerican,Hispanic,AfricanAmerican/Afro-Caribbean,Filipino,Seychelles,SouthAsian,Hispanic/Latino,EastAsian,AfricanBritish	10	rs1441264	LOC105370273 - NIPA2P5
14:79899454	European,AfricanAmerican,Filipino,Indian,AfricanAmerican/Afro-Caribbean,Hispanic/Latino,Hispanic,Seychelles,SouthAsian,Japanese	10	rs7141420	NRXN3
3:64718258	European,Filipino,AfricanAmerican/Afro-Caribbean,SouthAsian,Seychellois,AfricanAmerican,Indian,Hispanic/Latino,EastAsian,AfricanBritish	10	rs2371767	ADAMTS9-AS2
2:635721	European,Indian,Korean,AfricanAmerican,Jamaican,Filipino,AfricanAmerican/Afro-Caribbean,SouthAsian,Seychellois	9	rs6755502	LOC105373352 - TMEM18
1:109817192	Mexican,LatinAmerican/Hispanic,European,AfricanAmerican,HispanicAmerican,SouthAsian,Lebanese,EastAsian	8	rs7528419	CELSR2
1:184020945	European,AfricanAmerican,Filipino,Indian,AfricanAmerican/Afro-Caribbean,Hispanic/Latino,SouthAsian,Seychellois	8	rs2274432	TSEN15
7:2801803	European,AfricanAmerican,Filipino,Indian,Turkish,Moroccan,Surinamese,Chinese	8	rs798489	GNA12, AMZ1
20:45558831	European,Filipino,AfricanAmerican/Afro-Caribbean,SouthAsian,Seychellois,AfricanAmerican,Indian,Hispanic/Latino	8	rs6090583	EYA2
3:138108083	AfricanAmerican,European,Filipino,Indian,Hispanic/Latino,EastAsian,SouthAsian,AfricanBritish	8	rs1720825	MRAS
22:29449477	European,Filipino,AfricanAmerican/Afro-Caribbean,SouthAsian,Seychellois,AfricanAmerican,Indian,Hispanic/Latino	8	rs2294239	ZNRF3
2:27741237	European,Orcadian,AfricanAmerican,Hispanic,SaudiArabian,EastAsian,SouthAsian,Chinese	8	rs780094	GCKR

6:105378954	European,AfricanAmerican,Filipino,Indian,AfricanAmerican/Afro-Caribbean,SouthAsian,Seychellois,EastAsian	8	rs7759938	LOC100422315 - LINC00577
1:177852580	European,AfricanAmerican,Filipino,Indian,Japanese,AfricanAmerican/Afro-Caribbean,SouthAsian,Seychellois	8	rs633715	LOC101928778 - SEC16B
6:130345835	European,EastAsian,AfricanAmerican,Turkish,Moroccan,Surinamese,Chinese,Filipino	8	rs1415701	L3MBTL3
4:145574844	European,AfricanAmerican,Filipino,Indian,AfricanAmerican/Afro-Caribbean,SouthAsian,Seychellois,Hispanic/Latino	8	rs1812175	HHIP
11:116663707	Filipino,HanChinese,TaiwaneseHanChinese,EastAsian,European,Chinese,Yoruban	7	rs662799	ZNF259
12:112059557	European,AfricanAmerican,Asian,Hispanic,LatinAmerican,AfricanAmerican/Afro-Caribbean,African	7	rs11065979	ATXN2 - BRAP
8:126477978	Korean,EastAsian,European,AfricanAmerican/Afro-Caribbean,Indian,Filipino,Hispanic/Latino	7	rs2001945	TRIB1 - LOC105375746
6:26091179	European,AfricanAmerican,Hispanic,African,Asian,Brazilian,SouthAsian	7	rs1799945	HFE
22:29450923	AfricanAmerican,European,Filipino,Indian,AfricanAmerican/Afro-Caribbean,SouthAsian,Seychellois	7	rs2179129	ZNRF3
22:38595411	European,Indian,Korean,AfricanAmerican,Jamaican,EastAsian,Chinese	7	rs3761445	PLA2G6 - MAFF
19:45392254	European,Indian,Korean,AfricanAmerican,Jamaican,EastAsian,Latino	7	rs6857	PVRL2
3:64701146	European,AfricanAmerican/Afro-Caribbean,Indian,Filipino,Hispanic/Latino,SouthAsian,Seychellois	7	rs9860730	ADAMTS9-AS2
4:145601863	European,AfricanAmerican,Turkish,Moroccan,Surinamese,Chinese,Filipino	7	rs6537307	HHIP
8:41533514	European,AfricanAmerican,Turkish,Moroccan,Surinamese,Chinese,Filipino	7	rs13266210	ANK1
12:112072424	European,Hispanic/Latino,EastAsian,AfricanAmerican,SouthAsian,AfricanBritish	6	rs11065987	ATXN2 - BRAP
9:126590687	European,Hispanic/Latino,EastAsian,AfricanAmerican,SouthAsian,A	6	rs541160	DENND1A

	fricanBritish			
10:94462882	European,Japanese,EastAsian,SouthAsian,Mexican,Pakistani	6	rs1111875,CR072313	HHEX - EXOC6
2:622827	European,AfricanAmerican,Filipino,Indian,SouthAsian,EastAsian	6	rs2867125	LOC105373352 - TMEM18
7:46008110	European,African,Korean,Hispanic,AfricanAmerican,SouthAsian	6	rs11977526	LOC102723446
15:95267483	European,Hispanic/Latino,EastAsian,AfricanAmerican,SouthAsian,AfricanBritish	6	rs7181659	LOC105370986 - LOC105370987
19:7224431	European,EastAsian,Chinese,AfricanAmerican,Hispanic,SouthAsian	6	rs7248104	INSR, LOC107985283
7:129663496	European,AfricanAmerican,HispanicAmerican,SouthAsian,Lebanese,EastAsian	6	rs11556924	ZC3HC1
22:29451671	European,AfricanAmerican,Filipino,Indian,Hispanic,SouthAsian	6	rs4823006	ZNRF3
10:63462365	European,AfricanAmerican,Asian,Hispanic,LatinAmerican,AfricanAmerican/Afro-Caribbean	6	rs7922049	C10orf107
1:201801011	European,Hispanic/Latino,EastAsian,AfricanAmerican,SouthAsian,AfricanBritish	6	rs2494114	IPO9
6:26104632	European,AfricanAmerican,Asian,Hispanic,LatinAmerican,AfricanAmerican/Afro-Caribbean	6	rs198851	HIST1H4C - HIST1H1T
8:41509259	Japanese,EastAsian,European,SouthAsian,Mexican/Latino,Amish	6	rs12549902	NKX6-3 - ANK1
18:20735408	European,Filipino,AfricanAmerican/Afro-Caribbean,SouthAsian,Seychellois,EastAsian	6	rs4369779	CABLES1
1:201872264	AfricanAmerican,Filipino,Indian,European,Japanese,Mixed	6	rs2820315	LMOD1
4:156639888	European,AfricanAmerican,HispanicAmerican,SouthAsian,Lebanese,EastAsian	6	rs72689147	GUCY1A3
2:56111309	Korean,European,AfricanAmerican,Filipino,Indian,EastAsian	6	rs3791675	EFEMP1
5:95716722	European,Hispanic/Latino,EastAsian,AfricanAmerican,SouthAsian,AfricanBritish	6	rs7713317	LOC101929710
6:126752874	European,Filipino,AfricanAmerican/Afro-Caribbean,SouthAsian,Seychellois,Seychelles	6	rs1538170	CENPW

16:53824226	European,AfricanAmerican,Asian,Hispanic,LatinAmerican,African	6	rs62033406	FTO
15:66051299	European,Hispanic/Latino,EastAsian,AfricanAmerican,SouthAsian,AfricanBritish	6	rs12899850	DENND4A
12:111865049	European,AfricanAmerican,Asian,Hispanic,LatinAmerican,African	6	rs7310615	SH2B3
12:112273499	European,AfricanAmerican,Asian,Hispanic,LatinAmerican,African	6	rs11513729	ALDH2 - MAPKAPK5-AS1
1:109817590	AfricanAmerican,Afro-Caribbean,European,British,AfricanAmerican/Afro-Caribbean	5	rs12740374,CR105220	CELSR2
16:53800568	European,Filipino,AfricanAmerican/Afro-Caribbean,SouthAsian,Seychellois	5	rs9939973	FTO
20:6470094	European,Filipino,AfricanAmerican/Afro-Caribbean,SouthAsian,Seychellois	5	rs2326788	CASC20
11:116662579	HanChinese,Japanese,Korean,EastAsian	5	rs651821	ZNF259
8:41630405	European,EastAsian,Malay,SouthAsian,AfricanAmerican	5	rs4737009	ANK1
19:7184762	European,Filipino,AfricanAmerican/Afro-Caribbean,SouthAsian,Seychellois	5	rs891088	INSR
10:63467553	European,African,Asian,Hispanic,Brazilian	5	rs4590817	C10orf107
3:53125922	European,Filipino,AfricanAmerican/Afro-Caribbean,SouthAsian,Seychellois	5	rs11242	RFT1
16:53809247	European,Filipino,AfricanAmerican/Afro-Caribbean,SouthAsian,Seychellois	5	rs1121980	FTO
17:29630970	European,Filipino,AfricanAmerican/Afro-Caribbean,SouthAsian,Seychellois	5	rs3087591	EVI2B, NF1
8:19830921	European,Orcadian,AfricanAmerican,Afro-Caribbean,Japanese	5	rs10096633	LPL - LOC105379311
19:19407718	European,Indian,EastAsian,Chinese,Pakistani	5	rs10401969	SUGP1
7:2795957	European,Filipino,AfricanAmerican/Afro-Caribbean,SouthAsian,Seychellois	5	rs798497	GNA12, AMZ1
1:184018475	AfricanAmerican,European,Filipino,Indian,EastAsian	5	rs1926872	COLGALT2
12:111973358	European,AfricanAmerican,Asian,Hispanic,LatinAmerican	5	rs597808	ATXN2
2:277426	European,Mexican,LatinAmerican	5	rs780093	GCKR

03	/Hispanic,EastAsian,Chinese			
3:51208646	European,AfricanAmerican/Afro-Caribbean,Indian,Filipino,Hispanic/Latino	5	rs4378999	DOCK3
11:116607437	European,IndianAsian,Mexican,Indian,SaudiArabian	5	rs1558861	APOA5 - APOA4
1:11839923	European,AfricanAmerican,Asian,Hispanic,LatinAmerican	5	rs12561919	C1orf167
1:96893000	European,Filipino,AfricanAmerican/Afro-Caribbean,SouthAsian,Seychellois	5	rs11165623	UBE2WP1 - EEF1A1P11
3:64708114	European,Filipino,AfricanAmerican/Afro-Caribbean,SouthAsian,Seychellois	5	rs4132228	ADAMTS9-AS2
1:109818530	European,Orcadian,Finnish,SouthAsian,Japanese	5	rs646776	CELSR2 - PSRC1
5:95728898	European,Hispanic,Afro-Caribbean,Thai,FrenchCanadian	5	rs6235	LOC101929710, PCSK1
7:2789880	European,Filipino,AfricanAmerican/Afro-Caribbean,SouthAsian,Seychellois	5	rs798502	GNA12, AMZ1
7:2772431	European,Filipino,AfricanAmerican/Afro-Caribbean,SouthAsian,Seychellois	5	rs798528	AMZ1, GNA12
8:11255575	European,AfricanAmerican,Asian,Hispanic,LatinAmerican	5	rs9969423	FAM167A-AS1
4:145825151	European,Filipino,AfricanAmerican/Afro-Caribbean,SouthAsian,Seychellois	5	rs951252	HSPD1P5 - ANAPC10
8:19819439	European,Indian,Mexican,AfricanAmerican,Hispanic	5	rs326	LPL
8:126490972	European,Indian,HanChinese,EastAsian,Chinese	5	rs2954029	TRIB1 - LOC105375746
4:6289986	European,EastAsian,SouthAsian,Mexican,Pakistani	5	rs4458523	WFS1
3:138103927	AfricanAmerican,European,Filipino,Indian	4	rs1308362	MRAS
16:53825488	European,AfricanAmerican,Filipino,Indian	4	rs9941349	FTO
1:109817838	European,Indian,Mexican,LatinAmerican/Hispanic	4	rs660240	CELSR2
16:53830465	Sardinian,AfricanAmerican,Hispanic,European	4	rs9930506	FTO
15:58730498	Filipino,EastAsian,European,Chinese	4	rs588136	LIPC, LOC101928694

3:27446285	European,African,Asian,Hispanic	4	rs13063291	SLC4A7
3:11400249	European,SouthAsian,Hispanic,AfricanAmerican	4	rs2606736	ATG7
16:53799977	AfricanAmerican,European,Filipino,Indian	4	rs9930333	FTO
11:116652423	European,AfricanAmerican,Hispanic,Korean	4	rs6589566	ZNF259
1:96886267	AfricanAmerican,European,Filipino,Indian	4	rs587242	UBE2WP1 - EEF1A1P11
16:56997233	European,EastAsian,Chinese,Kuwaiti	4	rs1864163	CETP
19:33886354	AfricanAmerican,European,Filipino,Indian	4	rs7258031	PEPD
6:105364421	AfricanAmerican,European,Filipino,Indian	4	rs11156429	LOC100422315 - LINC00577
6:26107463	European,EastAsian,SouthAsian,AfricanAmerican	4	rs198846	HIST1H4C - HIST1H1T
8:41519248	SouthAsian,European,EastAsian,Pakistani	4	rs516946	ANK1
5:131770805	European,AfricanAmerican,Hispanic,SouthAsian	4	rs2188962	C5orf56
8:23595940	AfricanAmerican,Filipino,Indian,European	4	rs7004766	LOC107986930
5:131784393	European,SouthAsian,Hispanic,AfricanAmerican	4	rs12521868	C5orf56
4:145568352	European,AfricanAmerican,Filipino,Indian	4	rs7689420	HHIP
16:56989590	European,Finnish,EastAsian,Chinese	4	rs247616	HERPUD1, CETP
4:156635309	European,SouthAsian,Hispanic,AfricanAmerican	4	rs7692387	GUCY1A3
3:64701890	AfricanAmerican,European,Filipino,Indian	4	rs4504165	ADAMTS9-AS2
8:23593300	AfricanAmerican,Filipino,Indian,European	4	rs10101061	LOC107986930
16:56990716	AfricanAmerican,Hispanic,European	3	rs247617	HERPUD1, CETP
16:57005479	European,Orcadian,Mexican	3	rs1532624	CETP
6:52452585	EastAsian,European,Chinese	3	rs2239620	TRAM2-AS1 - LOC724104

8:19885726	EastAsian,European,Chinese	3	rs11984636	LPL - LOC105379311
8:126488250	EastAsian,European,Chinese	3	rs2980869	TRIB1 - LOC105375746
8:126495818	EastAsian,European,Chinese	3	rs10808546	TRIB1 - LOC105375746
10:17260290	European,EastAsian,Chinese	3	rs10904908	VIM-AS1
3:27472936	European,AfricanAmerican,Hispanic	3	rs3755652	SLC4A7
13:26781528	SouthAsian,European,EastAsian	3	rs10507349	RNF6
22:44377999	Mexican,LatinAmerican/Hispanic,European	3	rs2235776	SAMM50
8:126491733	Mexican,LatinAmerican/Hispanic,European	3	rs2954031	RP11-136O12.2
1:182150978	EastAsian,European,Chinese	3	rs1689797	ZNF648
1:63145439	EastAsian,European,Chinese	3	rs11208004	DOCK7
8:19819328	European,Indian,Japanese	3	rs325	LPL
7:116358044	European,EastAsian,Chinese	3	rs38855	MET
16:56995236	European,Filipino	3	rs1800775,CR000229	CETP
1:63094207	EastAsian,European,Chinese	3	rs11485618	DOCK7
19:19610596	SouthAsian,European,EastAsian	3	rs3794991	GATAD2A
16:57006590	Mexican,LatinAmerican/Hispanic,European	3	rs7499892	CETP
4:6270056	European,SouthAsian,EastAsian	3	rs4689388	WFS1
2:629244	European,EastAsian,SouthEastAsian	3	rs12463617	LOC105373352 - TMEM18
15:91521337	European,SouthAsian,EastAsian	3	rs8042680	PRC1, PRC1-AS1
11:61571478	EastAsian,European,Chinese	3	rs174550	FADS1
1:109822166	European,EastAsian,Chinese	3	rs599839	CELSR2 - PSRC1
1:150958836	European,EastAsian,Chinese	3	rs267733	ANXA9

13:32953388	European,EastAsian,Chinese	3	rs4942486	BRCA2
8:126481475	European,AfricanAmerican,Filipino	3	rs2980879	TRIB1 - LOC105375746
11:61549025	EastAsian,European,Chinese	3	rs174533	MYRF
1:63107526	EastAsian,European,Chinese	3	rs995000	DOCK7
8:126482621	European,EastAsian,Chinese	3	rs2954022	TRIB1 - LOC105375746
11:2213166	European,SouthAsian,EastAsian	3	rs7111341	TH - ASCL2
9:22051670	SouthAsian,European,EastAsian	3	rs944801	CDKN2B-AS1
1:63118196	European,Orcadian,Japanese	3	rs10889353	DOCK7
5:122476457	EastAsian,European,SouthAsian	3	rs13359291	PRDM6
8:19848080	AfricanAmerican,Hispanic,European	3	rs17410962	LPL - LOC105379311
11:61570783	European,EastAsian	2	rs174547	FADS1
2:21286057	European,Indian	2	rs515135	APOB - TDRD15
8:126482077	EastAsian,Japanese	2	rs2954021	TRIB1 - LOC105375746
4:6303022	European,Pakistani	2	rs1801214	WFS1
8:19824667	European,British	2	rs15285	LPL
19:11163601	European,Mixed	2	rs1122608	SMARCA4
1:63049551	HanChinese,European	2	rs11207995	DOCK7
8:19819724	HanChinese,European	2	rs328	LPL
8:19827848	European,Indian	2	rs10105606	LPL - LOC105379311
2:21241505	AfricanAmerican,Hispanic	2	rs12713956	APOB
8:126479362	SaudiArabian,Japanese	2	rs6982502	TRIB1 - LOC105375746
1:177913519	European,AfricanAmerican	2	rs10913469	SEC16B

3:64703394	AfricanAmerican,European	2	rs66815886	ADAMTS9-AS2
6:126851160	European,British	2	rs1490384	MIR588 - RNU6-200P
2:633660	African,European	2	rs62105306	LOC105373352 - TMEM18
2:27598097	AfricanAmerican,Hispanic	2	rs4665972	SNX17
9:22125503	European,HanChinese	2	rs1333049	CDKN2B-AS1 - DMRTA1
11:116647607	European,EastAsian	2	rs2160669	ZNF259
11:116584987	European,Indian	2	rs4938303	LOC101929011 - BUD13
3:64705365	European,Pakistani	2	rs6795735	ADAMTS9-AS2
11:61573684	European,Japanese	2	rs174551	FADS1
2:621558	Japanese,European	2	rs939584	LOC105373352 - TMEM18
16:53842908	Japanese,EastAsian	2	rs12149832	FTO
15:91544076	European,Pakistani	2	rs12899811	VPS33B
2:644953	European,AfricanAmerican	2	rs7561317	LOC105373352 - TMEM18
10:94465559	Mixed,European	2	rs5015480	HHEX - EXOC6
16:56988044	European	1	rs173539	HERPUD1, CETP
11:116660686	European	1	rs2266788	ZNF259
16:56987369	Japanese	1	rs56156922	HERPUD1, CETP
16:56994528	European	1	rs17231506,CR057853	CETP
11:116639104	European	1	rs10790162	BUD13
11:116652491	Japanese	1	rs7483863	ZNF259
11:116653296	European	1	rs2075290	ZNF259
16:56993886	European	1	rs821840	CETP

16:57002732	Japanese	1	rs9939224	CETP
3:64710253	European	1	rs918466	ADAMTS9-AS2
3:185317674	European	1	rs12374077	SENP2
1:16341649	European	1	rs1048238	HSPB7
2:28635740	European	1	rs7562	FOSL2
2:164444356	Japanese	1	rs77559470	LOC107985829 - FIGN
6:105407662	European	1	rs314277	LIN28B
12:102875569	European	1	rs35767	IGF1
17:68090207	European	1	rs11867479	KCNJ16
9:22124477	European	1	rs10757278	CDKN2B-AS1 - DMRTA1
10:94466439	Japanese	1	rs12219514	HHEX - EXOC6
2:21270554	European	1	rs585967	APOB - TDRD15
2:624034	European	1	rs6711012	LOC105373352 - TMEM18
3:135926622	European	1	rs645040	RPL31P23 - PCCB
8:126486409	European	1	rs17321515	TRIB1 - LOC105375746
2:21244000	European	1	rs10199768	APOB
1:55517301	Japanese	1	rs45576433	PCSK9
3:27457208	European	1	rs11716531	SLC4A7
12:9833628	European	1	rs3764021	CLEC2D
17:37745979	Kuwaiti	1	rs11654954	LOC105371770 - LOC105371771
5:74604742	HanChinese	1	rs6871667	ANKRD31 - HMGCR
19:10765	EastAsian	1	rs7250071	ILF3

819				
8:19824563	Japanese	1	rs1059611	LPL
6:126698719	European	1	rs9388489	CENPW
2:204738919	European	1	rs3087243	CTLA4 - LOC101927840
9:22088094	SaudiArab	1	rs10738607	CDKN2B-AS1
3:49913705	European	1	rs36022378	ACTBP13
12:48409054	European	1	rs12819124	LOC105369750
16:53845487	European	1	rs11642841	FTO
2:647861	European	1	rs4854349	LOC105373352 - TMEM18
20:6469596	European	1	rs17721822	CASC20
1:109819296	Mexican	1	rs3902354	CELSR2 - PSRC1
3:136107549	European	1	rs9880211	STAG1
2:630323	European	1	rs6725549	LOC105373352 - TMEM18
8:19832646	European	1	rs17482753	LPL - LOC105379311
2:27815510	European	1	rs13022873	ZNF512
2:27801759	European	1	rs1919128	C2orf16
7:117705257	Kuwaiti	1	rs39745	CTTNBP2 - LOC105375470
6:126712247	British	1	rs1578060	CENPW
8:126506694	European	1	rs112875651	TRIB1 - LOC105375746
14:68813115	European	1	rs1570106	RAD51B
1:93854517	Japanese	1	rs2114273	LOC100421273 - FNBP1L
2:214282	British	1	rs71435601	TDRD15 -

89				LOC105374317
16:53800754	European	1	rs9940128	FTO
2:624678	European	1	rs2903492	LOC105373352 - TMEM18
1:63085997	British	1	rs12239736	DOCK7
1:96944797	European	1	rs1555543	EEF1A1P11 - LOC105378866
17:37807698	Kuwaiti	1	rs9972882	STARD3
8:19849757	European	1	rs17091905	LPL - LOC105379311
8:126484526	European	1	rs2954026	TRIB1 - LOC105375746
15:73485160	European	1	rs11072405	NEO1
18:57852948	European	1	rs11152213	RNU4-17P - LOC342784
9:22077085	British	1	rs10811652	CDKN2B-AS1
11:116670676	European	1	rs6589567	APOA5 - APOA4
6:126990392	British	1	rs6918725	LOC105377992
19:11229218	EastAsian	1	rs2738452	LDLR
4:145565826	EastAsian	1	rs6845999	HHIP
8:126493746	European	1	rs2954033	TRIB1 - LOC105375746
2:27851918	European	1	rs3749147	GPN1
14:24830850	European	1	rs1950500	RIPK3 - NFATC4
3:53118739	European	1	rs2336725	RFT1
20:4098567	European	1	rs7273787	RPL21P2 - SMOX
16:81524274	European	1	rs12443634	CMIP

6:126752884	European	1	rs1538171	CENPW
12:90643524	European	1	rs10777237	LOC105369890 - LOC105369892
10:114808072	European	1	rs10885409	TCF7L2
3:53125585	European	1	rs2564921	RFT1
19:8644031	European	1	rs4072910	MYO1F - ADAMTS10
6:126966308	European	1	rs4549631	PRELID1P1 - LOC105377992
19:11224265	Japanese	1	rs5930	LDLR
6:126866133	European	1	rs1155939	MIR588 - RNU6-200P
4:145839264	European	1	rs4240326	HSPD1P5 - ANAPC10
11:116657561	Hispanic	1	rs3741298	ZNF259
4:145643079	European	1	rs6854783	HHIP
4:156638573	European	1	rs2306556	GUCY1A3
6:126717064	Mixed	1	rs1591805	CENPW
5:131686146	European	1	rs10058074	LOC553103
1:63078222	European	1	rs10889348	DOCK7
4:145650021	European	1	rs1492820	HHIP
8:19845376	EastAsian	1	rs7841189	LPL - LOC105379311
20:39672618	European	1	rs6029526	TOP1
5:74625487	Micronesian	1	rs7703051	ANKRD31 - HMGCR
1:182144119	European	1	rs2489279	ZNF648

11:61569830	European	1	rs174546	FADS1
2:169791438	European	1	rs552976	ABCB11
1:63049593	European	1	rs1748195	DOCK7
9:4290085	EastAsian	1	rs4237150	GLIS3
11:61579463	European	1	rs174554	FADS1
7:2763102	European	1	rs798544	AMZ1
7:2869985	European	1	rs1182188	GNA12
1:63049819	HanChinese	1	rs12042319	DOCK7
3:64702275	European	1	rs4616635	ADAMTS9-AS2
20:4101800	European	1	rs1741344	RPL21P2 - SMOX
1:109818306	European	1	rs629301	CELSR2
2:21288321	European	1	rs562338	APOB, LOC100287183
17:37832366	Kuwaiti	1	rs2934952	PGAP3
16:57000938	Mexican	1	rs118146573	CETP
22:38577515	Mixed	1	rs2277844	PLA2G6
2:21319016	British	1	rs672889	APOB - TDRD15
2:634905	European	1	rs6548238	LOC105373352 - TMEM18
9:22115286	Japanese	1	rs944797	CDKN2B-AS1
2:630075	British	1	rs73139123	LOC105373352 - TMEM18
8:19942181	Japanese	1	rs13263508	LOC105379311
15:74241506	European	1	rs750460	LOXL1
8:19824492	European	1	rs13702	LPL
9:22031005	European	1	rs7865618	CDKN2B-AS1
4:822203	EastAsian	1	rs2011962	PRKG2 -

24				RNU5A-2P
16:56987765	European	1	rs56228609	HERPUD1, CETP
16:56993161	European	1	rs12149545	CETP
8:126500031	European	1	rs28601761	RP11-136O12.2
16:56999328	Multi-ethnic cohort but largely European	1	rs11508026	CETP
16:56996211	European	1	rs711752	CETP
19:11227480	European	1	rs2738447	LDLR
18:57854694	European	1	rs34633411	RNU4-17P - LOC342784
2:21419650	European,Mixed	2	rs10221742	TDRD15 - LOC105374317
20:47456856	European, Mixed	2	rs56313611	LOC105372648
2:21291529	Multi-ethnic cohort but largely European	1	rs668948	APOB, LOC100287183
5:74262162	Multi-ethnic cohort but largely European	1	rs4703642	LOC105379040
3:135888642	European, Mixed	2	rs73222236	MSL2
8:126500350	European, Mixed	2	rs8180991	TRIB1 - LOC105375746
9:124412948	European, Mixed	2	rs10818576	DAB2IP
1:109821511	European	1	rs602633	CELSR2 - PSRC1
8:19843171	European, Mixed	2	rs17091891	LPL - LOC105379311
1:169094459	European, Mixed	2	rs1892094	ATP1B1
9:124420173	Mixed	1	rs885150	DAB2IP
